# Preparation and characterization of poly (ethersulfone) nanofiltration membranes for amoxicillin removal from contaminated water

**DOI:** 10.1186/2052-336X-12-18

**Published:** 2014-01-08

**Authors:** Maryam Omidvar, Seyed mahmoud Mousavi, Mohammad Soltanieh, Ali Akbar Safekordi

**Affiliations:** 1 Department of Chemical Engineering, Science and Research Branch, Islamic Azad University, Tehran, Iran; 2 Department of Chemical Engineering, Faculty of Engineering, Ferdowsi University of Mashhad, Mashhad, Iran

**Keywords:** Poly(ethersulfone), Membrane, Surfactant, Amoxicillin, Separation

## Abstract

Nowadays, antibiotics such as amoxicillin have been entered in water bodies. Nanofiltration has been proposed as an attractive technology for removal of antibiotics from aquatic environment instead of conventional wastewater treatment. In this paper, novel asymmetric flat sheet nanofiltration membranes were prepared via immersion precipitation technique and by using the poly(ethersulfone)/Brij^®^S100/Poly(vinylpirrolidone)/1-methyl-2-pyrolidone casting solutions. The effect of addition of Brij^®^S100 as a non-ionic surfactant additive as well as concentration of poly (ethersulfone) on morphology, wettability, pure water flux and rejection of amoxicillin were studied using the scanning electron microscopy, water contact angle apparatus and experimental set-up. The results indicated that the addition of Brij^®^S100 to the casting solutions resulted in the formation of membranes with higher hydrophilicity and relatively noticeable rejection of amoxicillin up to 99% in comparison with unmodified poly(ethersulfone) membrane. Contrary to amoxicillin rejection, pure water flux was decreased when higher poly(ethersulfone) concentration was employed.

## Introduction

Among all the pharmaceutical drugs that cause contamination of the environment, antibiotics occupy an important place due to their high consumption rates in both veterinary and human medicine [1]. Antibiotics as an important group of pharmaceutically active compounds (PhACs) were first produced in early 1940s and widely used in fighting against infectious bacteria and fungi [2]. Recently, antibiotics were quantified in hospital sewage water and wastewater, in rivers and in wastewater treatment plants (WTPs) [3].

The presence of antibiotics in the aquatic environment has created two issues. The immediate concern is the potential toxicity to aquatic organisms, and also to humans through drinking water. In addition, there is growing alarm that release of antibiotics to the environment contributes to the emergence of strains of disease-causing bacteria, resistant to high doses of these drugs [2]. Consequently, removal of antibiotics before they enter the aquatic environment, as well as for water reuse is very pertinent [2]. The molecular mass (MW) of antibiotics are in the range of 200 to 1,200 Daltons, coincident with the range of molecular mass cutoffs of NF membranes [4]. Membrane filtration using nanofiltration (NF) and reverse osmosis (RO) membranes is shown to be one of the most promising techniques for the removal of antibiotics [5].

There are several studies reported using NF as a tool for removal of pharmaceutical substances such as antibiotics [5-10].

Fouling in pressure-driven membrane processes like NF is a key design and operational concern; thus several control strategies have evolved to minimize its occurrence and impact. Fouling reduction involves one or a combination of three approaches viz. feed pre-treatment, controlling the system hydrodynamics and modifying the membrane characteristics [11]. Many investigations have demonstrated that increasing membrane surface hydrophilicity could effectively inhibit membrane fouling [12]. Therefore membrane with hydrophilic characteristics has drawn considerable attention in practical use in recent years because of its better fouling resistance [13].

Poly(ethersulfone) (PES), a transparent and amorphous polymer, is well-known due to its excellent heat deflection temperature, excellent toughness, dimensional stability, and resistance to steam, boiling water and mineral acids. Its other desirable properties include thermal stability, creep resistance, inherent flame resistance, and status as an approved material for use in food, water and medical applications. This polymer demonstrates moderate chemical resistance against many alkalis, and exhibits excellent biology and blood compatibility. All these properties make PES as an attractive material for membrane preparation. Its amorphous phase provides membrane flexibility while the crystalline phase provides the desired thermal stability [14]. The main disadvantage of PES membrane is the low hydrophilicity of the prepared membrane. Membrane surface properties often cause intense fouling when solutions containing substances like proteins are filtered. Therefore the modification of PES membrane is necessary for reducing the membrane fouling [14].

A promising in situ membrane surface modification approach can be obtained by addition of hydrophilic additives to the membrane casting solution. To improve the performance of PES membrane, researchers investigated the effect of some surfactants such as tetronic 1307 [15], sodium dodecyl sulphate (SDS), cetyle three methyl ammonium bromide (CTAB), triton x-100 [16] and tween 80 [17] on the properties and performance of PES membranes. They found out that addition of surfactant to the casting solution increased porosity of the membrane support layer and enhanced pure water permeability through the membranes. Surfactants constitute the most important group of detergents which are generally surface active agents. They are comprised of a hydrophobic portion attached to a hydrophilic functional group. Surfactants can be categorized according to the charge present in the hydrophilic portion of the molecule (after dissociation in the aqueous solution): anionic, cationic, non-ionic and zwitterionic surfactants [18,19].

There has been no prior study on the effect of Brij^®^S100 surfactant as a hydrophilic additive in order to improve the hydrophilic property of the PES nanofiltration membranes. As such, this research work investigates the preparation and characterization of these improved PES membranes. Membrane performance was evaluated in terms of concentrations of Brij^®^S100, PES and amoxicillin.

## Materials and methods

### Materials

**Figure 1:**
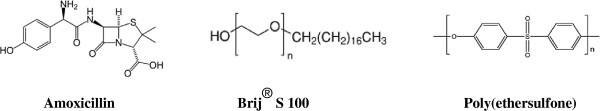
**Chemical structures of PES, Brij**^**^®^**^**S100 and amoxicillin.**

Poly(vinylpirrolidone) (PVP) with molecular weight of 40000 g/mol purchased from Merck was used as pore former. 1-methyl-2-pyrolidone (NMP), procured from Merck, and distilled water were applied as solvent and non-solvent, respectively. PES (Ultrason E6020P) with molecular weight of 58000gr/mol supplied from BASF was used as polymer in the casting solution. Brij^®^S100 (poly(oxyethylene (100) stearyl ether)) with the HLB = 18 purchased from Aldrich was applied as surfactant. Amoxicillin (pKa = 2.7) [6] was obtained from Dana pharmaceutical company. The chemical structure of PES, Brij^®^S100 and amoxicillin are illustrated in Figure 1. *N, N*-dimethyl-p-phenylenediamine, potassium hexacynoferrate (III), NH_3_ and NaOH were bought from Merck.

### Preparation of the membrane

Membranes were prepared by phase inversion method. PVP and Brij^®^S100 were added to the homogeneous solution of PES in NMP and mixed by stirring for 12 h at room temperature of 25 ± 2°C. The stirring was carried out at 200 rpm. Final prepared homogeneous solution was cast using a film applicator with 300 μm clearance gap on a glass plate and then moved to the non-solvent bath, distilled water at 0°C, for immersion precipitation step. After primary phase separation and formation of the membrane, it was stored in the water for 24 h to allow water soluble components to be leached out. At the final stage, the membrane was dried by placing it between two sheets of filter paper for 24 h at room temperature of 25 ± 2°C. Composition of the casting solutions is shown in Table 1.

**Table 1:** Composition of PES casting solution

**Membrane**	**PES (wt. %)**	**PVP (wt. %)**	**Brij**^**^®^**^**S100 (wt. %)**
M1	21	2	0
M2	21	2	2
M3	21	2	4
M4	21	2	6
M5	17	2	0
M6	17	2	2
M7	17	2	4
M8	17	2	6

### Membrane characterization

#### Membranes test by an experimental setup

**Figure 2:**
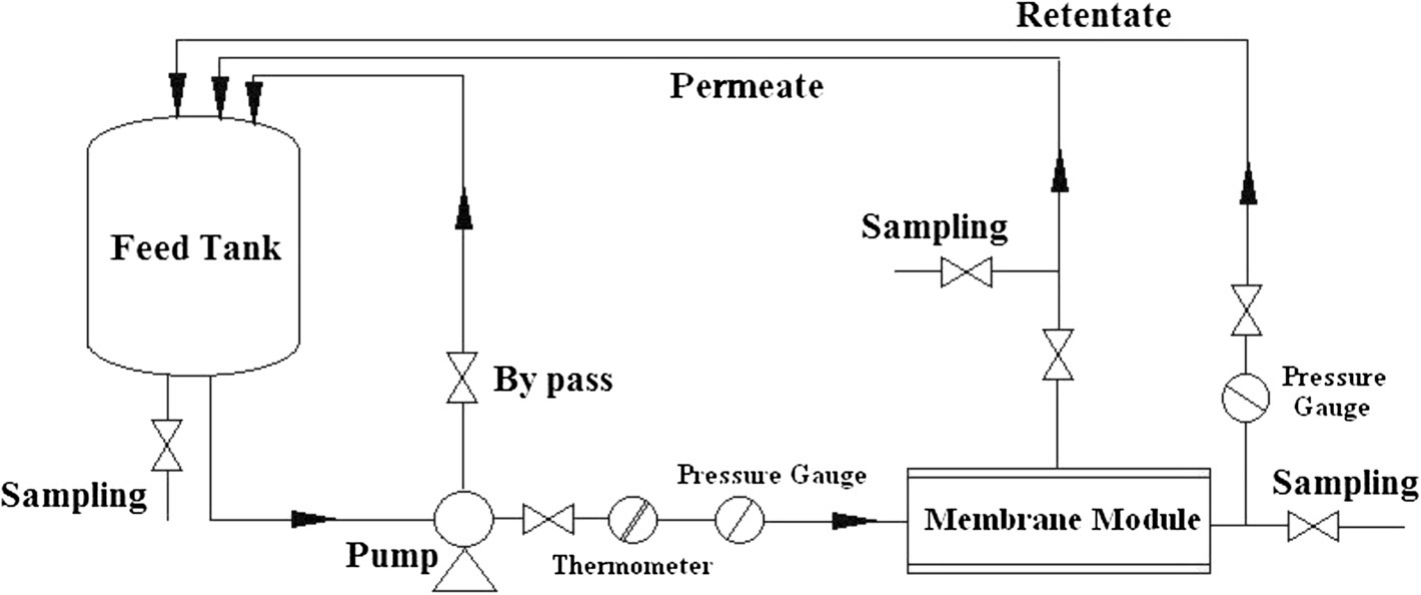
Schematic diagram of experimental set up.

The filtration experiments were conducted using a laboratory-scale membrane test unit which mainly consisted of feed tank, pump and membrane module (Figure 2). The effective membrane area in the module is 57 cm^2^. Full circulation mode was used during the experiments i.e. the retentate and permeate were returned to the feed tank in order to maintain constant concentration. All experiments were carried out at room temperature (25 ± 2°C). Pure water flux (PWF) and amoxicillin rejection were determined at a transmembrane pressure of 1 MPa. PWF was calculated using the following equation [9]:

(1) PWF=Q/A.Δt

Where Q is the quantity of permeate (L), A is the effective membrane area (m^2^) and Δt is the sampling time (h). Distilled water was used for the preparation of all stock solutions and membrane performance experiments. The treatment experiments were done at amoxicillin concentrations of 20 and 400 ppm for investigation of the effect of amoxicillin concentration in the feed on the performance of the membranes in amoxicillin separation. Amoxicillin rejection was calculated by the following equation [6]:

(2) $$R\;\left(\%\right)=\left(1-C_{p}/C_{f}\right)\times \;100$$

Where R is the rejection (%), and C_f_ and C_p_ are the solute concentration in feed and permeate samples, respectively. Amoxicillin concentration in the samples was determined by reacting amoxicillin with N, N-dimethyl-*p*-phenylenediamine in the presence of potassium hexacynoferrate (III) in an alkaline medium. The absorbance of the blue water-soluble reaction product was measured at 660 nm, using a UV–vis Spectrophotometer (T60, China) [20].

#### Scanning electron microscopy (SEM)

Membrane structure was examined by a scanning electron microscope (KYKY-EM 3200, China). To obtain a generally consistent and clean cut, membrane samples were held under liquid nitrogen and then snapped by flexing in one direction until it broke. After sputtering with gold, they were viewed with the microscope at 25 KV.

#### Contact angle

To determine the hydrophilicity of a membrane, the contact angle between a drop of distilled water and the membrane surface was measured at room temperature of 25 ± 2°C, using a contact angle measuring instrument (G10, KRUSS, Germany).

## Results and discussion

### Morphological studies of prepared membranes

#### Effect of Brij^®^S100 concentration

**Figure 3:**
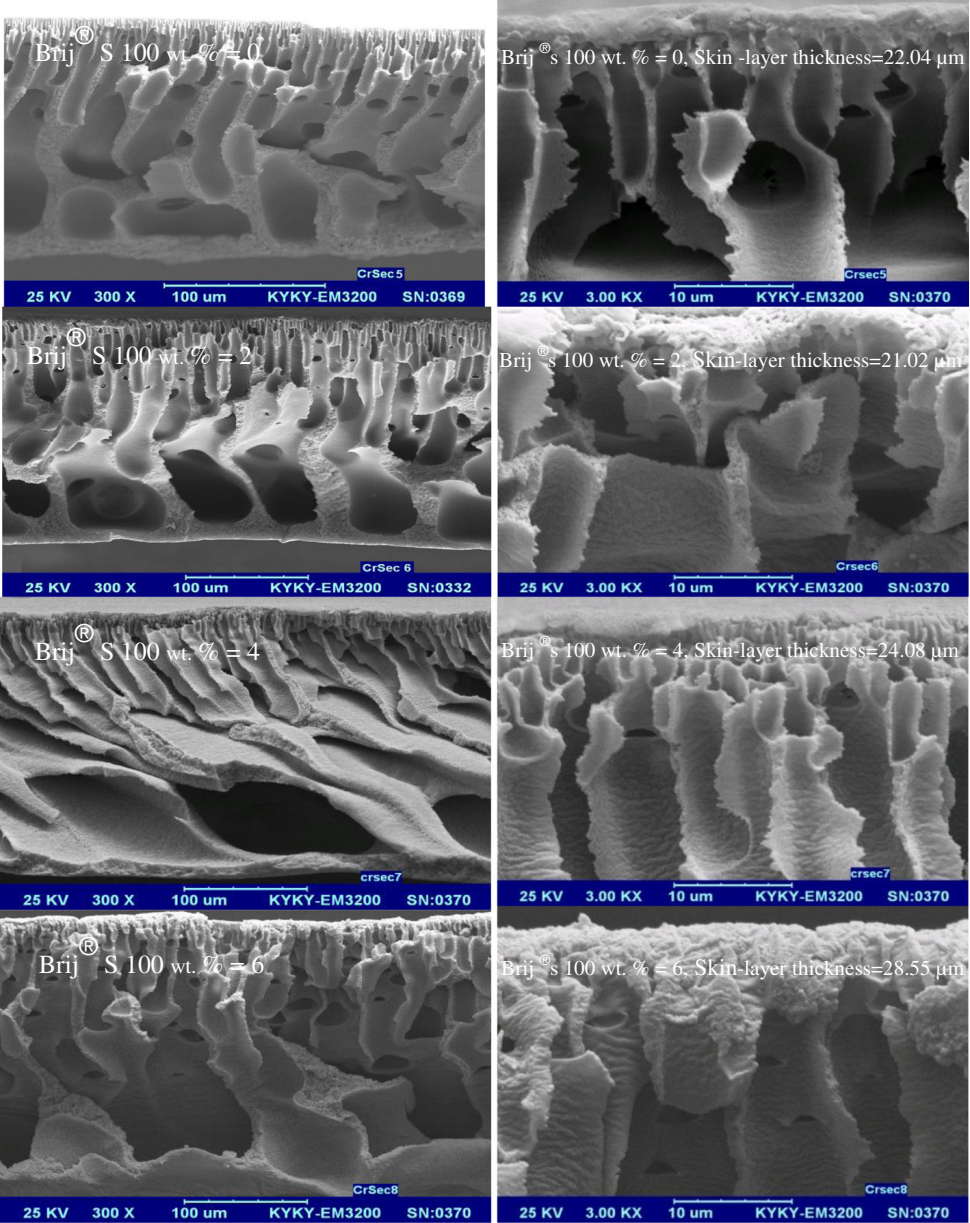
SEM cross-sectional images of the prepared membranes with 17 wt. % PES with two magnifications.

**Figure 4:**
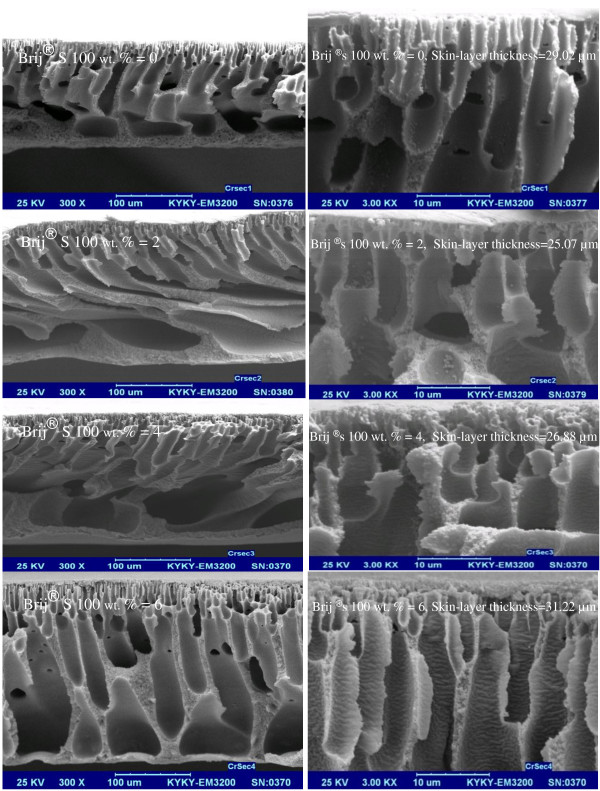
SEM cross-sectional images of the prepared membranes with 21 wt. % PES with two magnifications.

Cross-sectional SEM images of membranes with two different PES concentrations of 17 wt % and 21 wt % are shown in Figures 3 and 4, respectively. As observed from the images, the initial increase in Brij^®^S100 from 0 wt % to 2 wt % results in the formation of a more porous structure with larger voids in the top layer and sub-layer. However further increase in Brij^®^S100 concentration from 2 to 4 and 6 wt. % results in formation of denser structures. Formation of thicker dense top layer observed in Figures 3 and 4 due to mentioned increase in Brij^®^S100 concentration from 2 to 4 and 6 wt. % confirms above claim about formation of denser structures. According to Figures 3 and 4 membrane prepared with no Brij^®^S100 in the casting solution has thick dense top layer. A small amount of Brij^®^S100 (2 wt. %) in the casting solution changes the membrane morphology from a thick dense top layer to a thin dense top layer. However, more increase in Brij^®^S100\concentration in the casting solution from 2 to 4 and 6 wt. % causes formation of denser top-layer.

**Figure 5:**
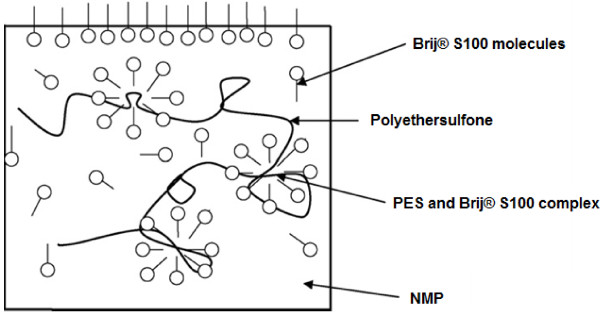
The effect of Brij^®^S100 on the formation of PES membrane by phase inversion.

Details of membrane formation mechanism were explained by Saljoughi and coworkers [21-24]. NMP (solvent) is hydrophilic and Brij^®^S100 is amphiphilic (i.e. with hydrophilic head and hydrophobic tail) and thus a layer of Brij^®^S100 molecules is formed on the surface of the casting film. This layer decreases surface tension and consequently facilitates water permeation into the casting solution [21,25]. On the other hand PES is relatively hydrophobic. The Brij^®^S100 molecules and PES tend to form a micelle-like complex in the solution (Figure 5). The formation of this micelle decreases the interaction between polymer chains [16]. Both phenomena result in instantaneous demixing in the coagulant bath [22-24]. As a result of the fact the growth of skin layer is reduced and formation of finger-like pores in the support is improved. Thus, primary increase of the Brij^®^S100 concentration up to 2 wt. % leads to formation of larger macrovoids and more porous structures. However, with further increase in Brij^®^S100 concentration (from2 wt. % to 4 and 6 wt. %) and because of the importance of viscosity effects, delayed demixing is preferred over instantaneous demixing. So higher concentrations of Brij^®^S100 can lead to the suppression of macrovoids and formation of denser structures [24]. Hence, it can be concluded that addition of hydrophilic additives such as Brij^®^S100 to the casting solution has a dual effect on the membrane morphology. In fact, the final membrane structure depends on the superiority of instantaneous or delayed demixing that both, as mentioned before, come from presence of Brij^®^S100 in the cast solution film [24]. According to Figures 3 and 4, increasing Brij^®^S100 concentration initially up to 2 wt. % causes formation of macrovoids and more porous structures. However, further increase of Brij^®^S100 concentration results in suppression of macrovoids and formation of denser structures.

#### Effect of PES concentration

The effect of variation of PES concentration on the membrane morphology is detected by comparison between Figures 3 and 4 as observed increase in PES concentration from 17 to 21wt. % results in the formation of smaller macrovoids and increase of thickness of the membrane top layer. Increase in the PES concentration from17 wt. % to 21 wt. % results in noticeable increase in viscosity values and consequently reduces mutual diffusivities between the nonsolvent (water) and solvent (NMP) in the system during solidification of the casting solution. Thus, using higher values of PES concentration, the precipitation process is stopped after a longer time and this leads to preparation of denser membranes [26,27].

### Contact angle

**Figure 6:**
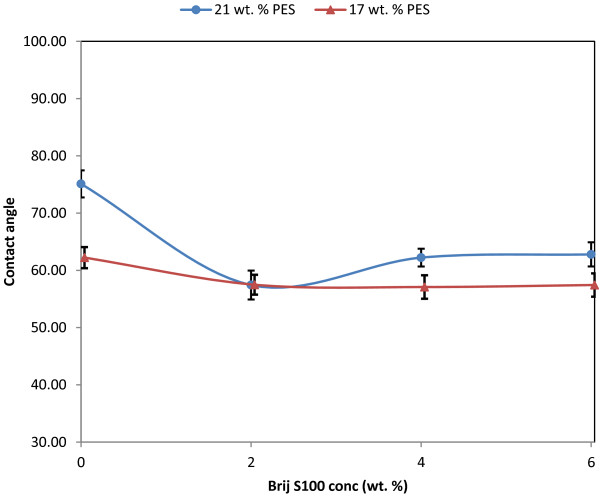
Contact angle vs. Brij^®^S100 concentration.

A hydrophilic membrane surface has a smaller contact angle than a hydrophobic membrane surface [28]. Figure 6 shows the measured contact angles for the membranes prepared from 17 wt. % and 21 wt. % of PES in the casting solutions. The membranes with higher PES concentration of 21 wt % showed a greater contact angle than the 17 wt % PES membranes. The effect of polymer concentration on the hydrophilicity of the membrane is due to the resultant pore size and porosity [29].

Susanto and Ulbricht [30] determined that a PES membrane without an additive had a lower contact angle than that typically measured for a non-porous PES film. They state that this is due to the porous structure in the outer membrane surface. Therefore, care should be taken to interpret the contact angle results because wettability is influenced not only by membrane material but also by the surface porosity. Thus the greater contact angle of the 21 wt % PES membranes can be attributed to the decreased surface porosity.

In fact higher porosity of membrane surface can reduce the contact angle of water drops on membrane surface. In addition, Figure 6 shows that the addition of Brij^®^S100 decreases the contact angle and hence increases hydrophilicity of the membranes. This can be attributed to the hydrophilic head of Brij^®^S100.

### PWF

**Figure 7:**
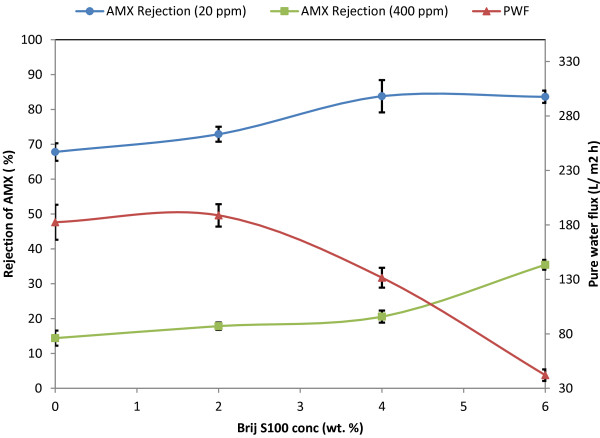
PWF and Rejection of amoxicillin (AMX) vs. Brij^®^S100 concentration for 17 wt. % PES.

**Figure 8:**
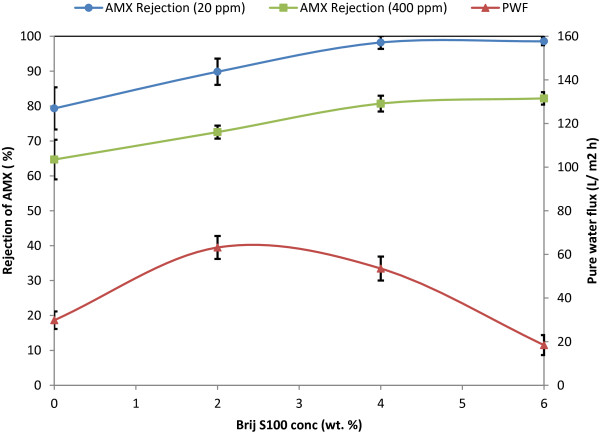
PWF and Rejection of amoxicillin (AMX) vs. Brij^®^S100 concentration for 21 wt. % PES.

The PWF results against various concentrations of PES polymer and Brij^®^S100 additive have been shown in Figures 7 and 8*.* As shown, the PWF through the membranes shows a nonlinear relationship with the Brij^®^S100 concentration. The PWF increases and reaches a maximum with the addition of 2 wt. % of Brij^®^S100 and then decreases with further addition of Brij^®^S100 in the casting solution. Also reduction of PWF with increase of PES concentration from 17 wt. % to 21 wt. % is noticeable. The changes imposed on membrane structure and properties after addition of Brij^®^S100 and variation of PES concentrations are major reasons for PWF changes. At first, it should be noted that PWF highly depends on membrane porosity and particularly the thickness and porosity of top layer [24,31]. Above results of PWF are in agreement with trend observed in SEM images. As mentioned before and observed in Figures 3 and 4, the initial increase in Brij^®^S100 concentration from 0 to 2 wt. % results in reduction of top layer thickness and consequently reduces resistance against the water permeation. However further increase in Brij^®^S100 concentration from 2 to 4 and 6 wt. % results in formation of thicker dense top layer and consequently, intensifies resistance against water permeation. Also comparison between Figures 3 and 4 reveals higher porosity and consequently less resistance against water permeation in the membranes prepared from 17 wt. % of PES in comparison with the membranes prepared from 21 wt. % of PES.

### Rejection of amoxicillin

The rejection results of the solutions containing amoxicillin using the prepared membranes are depicted in Figures 7 and 8.

#### Effect of Brij^®^S100 and PES concentration

As observed in Figures 7 and 8, increase in PES concentration from 17 wt. % to 21 wt. % along with increase in Brij^®^S100 concentration from 0 wt. % to 6 wt. %, results in higher rejection of amoxicillin. So that M5 membrane, prepared from 17 wt. % PES and without addition of Brij^®^S100 in the casting solution showed the lowest rejection of amoxicillin, whereas maximum rejection of amoxicillin was obtained for M4, the membrane which contains maximum concentration of PES and Brij^®^S100 in its casting solution, i.e. 21 wt. % and 6 wt. %, respectively. These observations are in agreement with the literature [19,21,32].

Generally rejection of organic compounds by NF membranes is performed based on the size exclusion (steric hindrance), electrostatic charge repulsion and adsorption on the membrane surface which are related to the membrane and solute properties and solution conditions [19,32]. Because some pharmaceutical compounds such as amoxicillin are hydrophilic [33] they are not mostly adsorbed on the membrane surface. Consequently, the removal can occur through steady-state rejection due to either steric effects for uncharged solutes or combined steric and electrostatic effects for charged solutes.

By comparison between the rejection values obtained from Figures 7 and 8 with SEM images, it is found out that the main mechanism governing separation of amoxicillin is steric hindrance, because generally, the membranes with denser structures i.e. ones prepared with higher concentration of PES and Brij^®^S100 show higher rejection of amoxicillin. Nghiem *et al.*[34] reported that the retention of pharmaceuticals by a tight NF membrane is dominated by steric exclusion, whereas both electrostatic repulsion and steric exclusion govern the retention of ionizable pharmaceuticals by a loose NF membrane. This is fully in line with our results and interpretation.

#### Effect of amoxicillin concentration

As shown in Figures 7 and 8, the increase in amoxicillin concentration results in the reduction of amoxicillin rejection. This may be due to concentration polarization. Shahtalebi *et al.*[9]. investigated the effect of amoxicillin concentration on the performance of commercial NF membranes. They discovered that the increase of amoxicillin concentration results in lower flux. They found out that the concentration polarization occurs in the membrane separation process and has an important influence on the membrane separation performance. When the phenomenon of concentration polarization takes place, a layer is formed at the membrane-liquid interface. The concentration of solute in this layer is higher than that of the solution bulk on the high pressure side. The concentration polarization layer holds up the transport through the membrane, because the increase in osmotic pressure reduces the driving force of mass transfer. Consequently, flux decreases [9]. Also the rejection of amoxicillin reduces.

## Conclusions

The modification of PES nanofiltration membrane was carried out by the addition of different concentrations of Brij^®^S100 hydrophilic surfactant into the casting solution. The membranes performance was studied in terms of PWF and rejection of amoxicillin. The addition of 2 wt. % Brij^®^S100 to the casting solution increased the formation of macrovoid in the sub-layer of these membranes and consequently resulted in increasing PWF. With further increase in Brij^®^S100 concentrations from 2 wt. % to 6 wt. % and because of the importance of viscosity effects, the membranes structure, particularly top-layer zone, become denser and consequently PWF decreased. The morphological and experimental studies revealed that the addition of Brij^®^S100 to the casting solutions resulted in the formation of membranes with higher hydrophilicity and rejection of amoxicillin in comparison with net PES membrane. Lower PES concentrations resulted in the simultaneous increase in PWF and the transmission of amoxicillin through the membranes. Also amoxicillin rejection was decreased by increasing the concentration of amoxicillin in feed.

## Competing interests

The authors declare that they have no competing interests.

## Authors’ contributions

MO participated in the acquisition, analysis, and interpretation of data and helped to draft the manuscript. SMM and MS supervised the study in all steps (acquisition, analysis, and interpretation of data) and has been consulted by AAS. All authors read and approved the final manuscript.
